# Supplementary material for: Risk Assessment of (Herbal) Teas Containing Pyrrolizidine Alkaloids (PAs) Based on Margin of Exposure Approach and Relative Potency (REP) Factors
Source: Foods. 2022 Sep 21;11(19):2946. doi: 10.3390/foods11192946 (PMC9564199; doi:10.3390/foods11192946)
Supplement: Supplementary file 1 [file foods-11-02946-s001.zip › foods-1899434-Supporting data 1.pdf]

Supporting data 1 to the Manuscript:

**Risk assessment of (herbal) teas containing pyrrolizidine alkaloids (PAs) based on margin of exposure approach and relative potency (REP) factors**

Lu Chen[2,a], Qian Zhang[1,a], Ziwei Yi[1], Yu Chen[1], Weihan Xiao[1], Dan Su[3], Wenbiao Shi[1]*

[1] Department of Nutrition and Health, Chinese Agriculture University, Beijing 100091, China

[2] Institute of Quality Standards and Testing Technology for Agro-Products, Chinese Academy of Agricultural Sciences; Key Laboratory of Agri-food Quality and Safety, Ministry of Agriculture and Rural Affairs, Beijing 100081, China

[3] Department of Chemistry and Chemical Biology, Cornell University, Ithaca, NY 14850, USA

[a] These authors contributed equally to this study and share first authorship

* Corresponding author: Wenbiao Shi

Department of Nutrition and Health, Chinese Agriculture University.

Beijing, 100083, China

Phone: +86 010 62738589

Email: wenbiao.shi@cau.edu.cn

## Supplemental tables

**Table S1.** Mass parameters for individual PA compounds.

| Compound | Precursor ion (m/z) | Cone voltage (V) | Product ion 1 (m/z) | Product ion 2 (m/z) | Product ion 3 (m/z) | RT (min) |
|---|---|---|---|---|---|---|
| 7-Acetylintermedine | 342.2 | 30 | 120 | 180 | 94 | 8.80 |
| 7-Acetylintermedine N-oxide | 358.2 | 30 | 137 | 214 | 180 | 5.90 |
| 7-Acetyllycopsamine | 342.2 | 30 | 120 | 180 | 94 | 8.85 |
| 7-Acetyllycopsamine N-oxide | 358.2 | 30 | 137 | 214 | 180 | 5.90 |
| Acetylpetasitenine | 424.2 | 30 | 122 | 168 | 150 | 9.10 |
| Adonifoline | 366.2 | 40 | 94 | 338 | 120 | 5.55 |
| Amabiline | 284.2 | 30 | 122 | 140 | 94 | 3.30 |
| Atropine | 290.2 | 40 | 124 | 93 | 94 | 4.15 |
| Echimidine | 398.2 | 30 | 120 | 220 | 83 | 10.55 |
| Echimidine N-oxide | 414.2 | 30 | 254 | 352 | 94 | 7.40 |
| Echinatine | 300.2 | 30 | 138 | 156 | 94 | 6.45 |
| Echinatine N-oxide | 316.2 | 30 | 111 | 172 | 94 | 3.75 |
| Erucifoline | 350.2 | 40 | 94 | 120 | 138 | 7.30 |
| Erucifoline N-oxide | 366.2 | 40 | 94 | 118 | 120 | 3.25 |
| Europine | 330.2 | 30 | 94 | 138 | 156 | 6.15 |
| Europine N-oxide | 346.2 | 30 | 172 | 111 | 256 | 3.55 |
| Florosenine | 424.2 | 30 | 122 | 168 | 150 | 8.40 |
| Heliosupine | 398.2 | 30 | 120 | 220 | 336 | 10.40 |
| Heliosupine N-oxide | 414.2 | 30 | 94 | 254 | 138 | 7.10 |
| Heliotrine | 314.2 | 30 | 138 | 156 | 94 | 8.10 |
| Heliotrine N-oxide | 330.2 | 30 | 111 | 172 | 94 | 5.50 |
| Indicine N-oxide | 316.2 | 30 | 94 | 172 | 111 | 3.55 |
| Integerrimine | 336.2 | 40 | 94 | 120 | 138 | 9.95 |
| Integerrimine N-oxide | 352.2 | 40 | 94 | 120 | 136 | 6.50 |
| Intermedine | 300.2 | 30 | 94 | 156 | 138 | 5.45 |
| Intermedine N-oxide | 316.2 | 30 | 94 | 172 | 111 | 3.40 |
| Jacobine | 352.2 | 40 | 120 | 155 | 94 | 7.65 |
| Jacobine N-oxide | 368.2 | 40 | 120 | 296 | 119 | 4.40 |
| Jacoline | 370.2 | 40 | 94 | 138 | 120 | 5.35 |
| Jacoline N-oxide | 386.2 | 40 | 94 | 120 | 138 | 2.60 |
| Jaconine | 388.2 | 40 | 94 | 120 | 138 | 8.75 |
| Jaconine N-oxide | 404.2 | 40 | 94 | 120 | 118 | 4.90 |
| Lasiocarpine | 412.2 | 30 | 120 | 220 | 336 | 11.25 |
| Lasiocarpine N-oxide | 428.2 | 30 | 138 | 254 | 94 | 8.25 |
| Lycopsamine | 300.2 | 30 | 94 | 156 | 138 | 5.50 |

| | | | | | | |
|---|---|---|---|---|---|---|
| Lycopsamine N-oxide | 316.2 | 30 | 94 | 172 | 111 | 3.45 |
| Merenskine | 388.2 | 40 | 94 | 120 | 138 | 9.05 |
| Merenskine N-oxide | 404.2 | 40 | 94 | 120 | 118 | 5.10 |
| Merepoxine | 352.2 | 40 | 94 | 120 | 138 | 8.20 |
| Merepoxine N-oxide | 368.2 | 40 | 94 | 120 | 119 | 5.00 |
| Monocrotaline | 326.2 | 40 | 94 | 120 | 121 | 5.60 |
| Monocrotaline N-oxide | 342.2 | 40 | 120 | 137 | 94 | 2.45 |
| Neosenkirkine | 366.2 | 30 | 122 | 168 | 150 | 6.85 |
| Otosenine | 382.2 | 30 | 122 | 168 | 150 | 4.55 |
| Petasitenine | 382.2 | 30 | 122 | 168 | 150 | 5.90 |
| Retrorsine | 352.2 | 40 | 94 | 120 | 138 | 8.55 |
| Retrorsine N-oxide | 368.2 | 40 | 94 | 120 | 119 | 5.40 |
| Riddelliine | 350.2 | 40 | 94 | 120 | 138 | 7.80 |
| Riddelliine N-oxide | 366.2 | 40 | 94 | 118 | 120 | 4.50 |
| Rinderine | 300.2 | 30 | 138 | 156 | 94 | 6.60 |
| Rinderine N-oxide | 316.2 | 30 | 111 | 172 | 94 | 3.85 |
| Sceleratine | 370.2 | 40 | 94 | 138 | 120 | 5.75 |
| Sceleratine N-oxide | 386.2 | 40 | 94 | 120 | 138 | 2.80 |
| Scopolamine | 304.2 | 30 | 138 | 156 | 94 | 2.95 |
| Senecionine | 336.2 | 40 | 94 | 120 | 138 | 10.25 |
| Senecionine N-oxide | 352.2 | 40 | 94 | 120 | 136 | 6.70 |
| Seneciphylline | 334.2 | 40 | 120 | 138 | 94 | 9.25 |
| Seneciphylline N-oxide | 350.2 | 40 | 94 | 138 | 118 | 5.80 |
| Senecivernine | 336.2 | 40 | 94 | 120 | 138 | 10.40 |
| Senecivernine N-oxide | 352.2 | 40 | 94 | 120 | 136 | 6.75 |
| Senkirkine | 366.2 | 30 | 122 | 168 | 150 | 7.00 |
| Spartioidine | 334.2 | 40 | 120 | 138 | 94 | 8.95 |
| Spartioidine N-oxide | 350.2 | 40 | 94 | 120 | 118 | 5.70 |
| Supinine | 284.2 | 30 | 122 | 140 | 110 | 6.90 |
| Supinine N-oxide | 300.2 | 30 | 120 | 156 | 140 | 4.95 |
| Trichodesmine | 354.2 | 40 | 120 | 222 | 121 | 8.65 |
| Trichodesmine N-oxide | 370.2 | 40 | 137 | 238 | 94 | 5.30 |
| Usaramine | 352.2 | 40 | 94 | 120 | 138 | 8.30 |
| Usaramine N-oxide | 368.2 | 40 | 94 | 120 | 119 | 5.25 |

**Table S2.** The REP factors of individual PA and their *N*-ox congeners in this study according to Merz and Schrenk (2016) [1].

| PA and their *N*-ox congeners | REP factor | PA and their *N*-ox congeners | REP factor |
|---|---|---|---|
| 7-Acetylintermedine | 0.1 | Lycopsamine *N*-ox | 0.01 |
| 7-Acetyllycopsamine | 0.1 | Merenskine | 1 |
| 7-Acetyllycopsamine/intermedine *N*-ox | 0.1 | Merenskine *N*-ox | 1 |
| Acetylpetasitenine | 1 | Merepoxine | 1 |
| Adonifoline | 1 | Merepoxine *N*-ox | 1 |
| Amabiline | 0.01 | Monocrotaline | 1 |
| Atropine | 0 | Monocrotaline *N*-ox | 1 |
| Echimidine | 0.1 | Neosenkirkine | 1 |
| Echimidine *N*-ox | 0.1 | Otosenine | 1 |
| Echinatine | 0.3 | Petasitenine | 1 |
| Echinatine *N*-ox | 0.3 | Retrorsine | 1 |
| Erucifoline | 1 | Retrorsine *N*-ox | 1 |
| Erucifoline *N*-ox | 1 | Riddelliine | 1 |
| Europine | 0.3 | Riddelliine *N*-ox | 1 |
| Europine *N*-ox | 0.3 | Rinderine | 0.3 |
| Florosenine | 1 | Rinderine *N*-ox | 0.3 |
| Heliosupine | 1 | Sceleratine | 1 |
| Heliosupine *N*-ox | 1 | Sceleratine *N*-ox | 1 |
| Heliotrine | 0.3 | Scopolamine | 0 |
| Heliotrine *N*-ox | 0.3 | Senecionine | 1 |
| Indicine *N*-ox | 0.01 | Senecionine *N*-ox | 1 |
| Integerrimine | 1 | Seneciphylline | 1 |
| Integerrimine *N*-ox | 1 | Seneciphylline *N*-ox | 1 |
| Intermedine | 0.01 | Senecivernine | 1 |
| Intermedine *N*-ox | 0.01 | Senecivernine *N*-ox | 1 |
| Jacobine | 1 | Senkirkine | 1 |
| Jacobine *N*-ox | 1 | Spartioidine | 1 |
| Jacoline | 1 | Spartioidine *N*-ox | 1 |
| Jacoline *N*-ox | 1 | Supinine | 0.01 |
| Jaconine | 1 | Supinine *N*-ox | 0.01 |
| Jaconine *N*-ox | 1 | Trichodesmine | 1 |
| Lasiocarpine | 1 | Trichodesmine *N*-ox | 1 |
| Lasiocarpine *N*-ox | 1 | Usaramine | 1 |
| Lycopsamine | 0.01 | Usaramine *N*-ox | 1 |

**References**

1. Merz, K.-H.; Schrenk, D. Interim Relative Potency Factors for the Toxicological Risk Assessment of Pyrrolizidine Alkaloids in Food and Herbal Medicines. *Toxicol. Lett.* **2016**, *263*, 44–57, doi:10.1016/j.toxlet.2016.05.002.
